# The impact of perceived social support on sleep quality in a sample of patients undergoing hemodialysis in Somalia

**DOI:** 10.3389/fpsyt.2023.1108749

**Published:** 2023-03-06

**Authors:** Nur Adam Mohamed, Yusuf Abdirisak Mohamed, Asir Eraslan, Samet Kose

**Affiliations:** ^1^Department of Psychiatry and Behavioral Sciences, Mogadishu Somalia Turkey Recep Tayyip Erdogan Research and Training Hospital, Mogadishu, Somalia; ^2^Department of Urology, Mogadishu Somalia Turkey Recep Tayyip Erdogan Research and Training Hospital, Mogadishu, Somalia

**Keywords:** end-stage renal disease, hemodialysis, social support, quality of sleep, Somalia

## Abstract

**Objective:**

The main objective of the present study is to examine the relationship between perceived social support and the quality of sleep and to determine the predictors of sleep quality in a sample of patients undergoing hemodialysis (HD) in Somalia.

**Methods:**

A sample of 200 patients with end-stage renal disease (ESRD) who were undergoing hemodialysis treatment approximately two to three times a week were included. All participants were administered a sociodemographic data form, the Multidimensional Scale of Perceived Social Support (MSPSS), the Insomnia Severity Index (ISI), and the Pittsburgh Sleep Quality Index (PSQI). Patients undergoing HD for less than 3 months prior to the study date were excluded.

**Results:**

Of the patients undergoing hemodialysis, 200 patients aged between 18 and 68 years (mean = 52.29; SD = 14.13) gave consent and participated in the study. Sixty-three subjects (31.5%) reported poor sleep quality, defined as having a total PSQI score > 5. Forty-one subjects (20.5%) reported clinically significant (moderate-to-severe) insomnia. The majority of our patients undergoing HD reported remarkably high family support, but low friends and significant other support. Poor sleep quality significantly correlated with perceived friends’ support and perceived total social support. While perceived family support significantly correlated with both family income and the duration of chronic kidney disease (CKD), perceived friends’ support significantly correlated with age and family income. Hierarchical regression analyses showed that perceived family support and friends’ support were significant predictors of poor sleep quality. Perceived friends’ support was a significant predictor of insomnia severity. Perceived family support was a significant predictor of subjective sleep quality and sleep duration. Perceived friends’ support was a significant predictor of subjective sleep quality, sleep duration, sleep latency, sleep disturbance, and daytime dysfunction. Family income was a significant predictor of sleep duration. Age and gender were significant predictors of sleep efficiency. The duration of CKD and duration of HD were significant predictors of sleep disturbance.

**Conclusion:**

This present study has highlighted the value of family as a principal support system in Somalian culture. Understanding the impact of perceived social support on the quality of sleep in patients undergoing HD will help healthcare providers and social services to focus on and improve the social support systems of the patients as an integral part of their treatment.

## 1. Introduction

Maintenance hemodialysis is currently the standard treatment of choice for patients with end-stage renal disease (ESRD), and more than 3 million patients with ESRD worldwide receive this treatment. Many patients undergoing hemodialysis report a poor quality of sleep, and this can potentially predict their morbidity, mortality, and overall quality of life. According to the current research, 40–85% of patients undergoing hemodialysis report sleeping problems (1–3). Insomnia, restless legs syndrome (RLS), breathing problems during sleeping, and excessive daytime sleepiness (EDS) are most commonly reported in patients with ESRD undergoing HD (24). Sleep problems in patients undergoing HD have been linked to a variety of factors, such as behavioral disturbances, biological traits, medical comorbidities, treatment-related parameters, and psychosocial circumstances (4).

Several studies have shown that sleep was disturbed in patients with ESRD undergoing hemodialysis treatment (5–7). The underlying causes of sleep disturbances were reported to be multifactorial including electrolyte imbalances, uremia, erythropoietin deficiency-related anemia, and circadian rhythm disturbances due to melatonin release. Reduced quality of life in patients undergoing hemodialysis can also be caused by sleep disturbances, which result in poor daily life functioning, inability to care for one’s family needs, and inability to actively participate in social life (5–7).

Poor social support and a lack of communication with friends and family are some of the major challenges that patients with ESRD undergoing hemodialysis encounter (25). It was shown that the level of social support was crucial in a more efficient adaptation to the chronic nature of the illness, sleep problems, and potential complications encountered during the treatment (8). According to Cohen et al. (9), social support refers to the intricate network in which an individual might receive and provide assistance and have his/her emotional needs met. Social support supposedly creates a sense of physical and psychological wellbeing and has been shown to have a profound influence on the daily lives of patients undergoing HD (9, 10). Social support is often provided by family members, friends, and significant others and constitutes cognitive, emotional, and materials’ support supplied to the individual (9). Although the need for examining social support has been emphasized in patients undergoing hemodialysis, to the best of our knowledge, no studies have examined this relationship and the predictive capacity of social support on the quality of sleep as a measure of overall quality of life. This is also the first study in Africa to examine this crucial relationship.

In this present study, we aimed to examine the relationship between perceived social support and the quality of sleep and to determine the predictors of sleep quality in a sample of patients undergoing hemodialysis in Somalia.

## 2. Methods

The study design was cross-sectional and was conducted at the hemodialysis unit of Mogadishu Somalia–Turkey Recep Tayyip Erdogan Research and Training Hospital in Mogadishu, Somalia. The participants included 200 (83 women and 117 men) patients who were undergoing hemodialysis treatment approximately two to three times a week. Patients undergoing hemodialysis for less than 3 months prior to the study date were not included in the study. The study protocol was approved by the Hospital’s Ethics Review Board (MSTH/10515, Date: 05/30/2022). All participants were administered a sociodemographic data form, the Pittsburgh Sleep Quality Index (PSQI), the Insomnia Severity Index (ISI), and the Multidimensional Scale of Perceived Social Support (MSPSS).

### 2.1. Psychometric scales

#### 2.1.1. The Pittsburgh sleep quality index (PSQI)

The PSQI is a self-administered questionnaire created by Buysse et al. (11) that contains 19 self-rated questions and five questions that are rated by a bed partner or roommate. It evaluates patients’ perceptions of their sleep quality over the previous 4 weeks. In total, seven component scores are created by adding the 19 self-rating questions together: subjective sleep quality, sleep latency, sleep duration, sleep efficiency, sleep disruption, the usage of sleep drugs, and daytime dysfunction. Each component is graded on a scale of 0–3, with a score of “0″ indicating no difficulty, while a score of “3″ indicates great difficulty for each component. A total PSQI score will be generated from the seven component scores, and it will range from 0 to 21 points; higher values signify poorer sleep quality. Patients who obtain a global PSQI score of > 5 are referred to as “poor sleepers,” whereas patients who obtain a score of 5 are referred to as “good sleepers.” Although bed partner or roommate responses do not count in the overall PSQI total score, they can also be scored from 0 to 3 points depending on the severity of the symptom. Cronbach’s alpha of the PSQI of the original study (11) was reported as 0.83. The item-total score correlations ranged from 0.19 to 0.69. In this present study, Cronbach’s alpha of the PSQI for the present study sample was 0.85. The item-total score correlations ranged from 0.26 to 0.86, with an average of 0.66.

#### 2.1.2. The insomnia severity index (ISI)

The ISI is a seven-item self-reported questionnaire, created by Morin et al. (12) to evaluate the type, intensity, and effects of insomnia. The usual recall period is the “last month,” and the dimensions assessed are the severity of sleep onset, sleep maintenance, early morning awakening problems, sleep dissatisfaction, the interference of daytime functioning with sleep difficulties, the noticeability of sleep problems by others, and distress brought on by the lack of sleep. Each item is rated on a 5-point Likert scale (0 = no difficulty, 4 = very severe problem), which results in a total score that ranges from 0 to 28. The following categories of insomnia are used to interpret the overall score: no insomnia (0–7), sub-threshold insomnia (8–14), moderate insomnia (15–21), and severe insomnia (22–28). There are three versions available: patient, clinician, and significant other. Cronbach’s alpha of the ISI of the original study was reported as 0.74. The item-total score correlations ranged from 0.36 to 0.67, with an average of 0.54. In this present study, Cronbach’s alpha of the ISI for the present study sample was 0.74. The item-total score correlations ranged from 0.69 to 0.83, with an average of 0.74.

#### 2.1.3. The multidimensional scale of perceived social support (MSPSS)

The MSPSS is a self-rated measurement tool developed by Zimet et al. (13). It consists of 12 self-reported items that are intended to assess the degree of perceived social support from three different groups: family (items 3, 4, 8, and 11), friends (items 6, 7, 9, and 12), and significant others (items 1, 2, 5, and 10). Each response is given a Likert-type response score between 1 and 7, where 1 represents a very strong disagree and 7 represents a very strong agree. The results for each item are added together to produce a final score. The overall score ranges from 12 to 84, or it can be scored according to its subscales by combining the items in each subscale and then dividing by 4. A higher score indicates more social support than a person perceives. The subscales’ and dimensions’ range of possible scores is from 4 to 28. Cronbach’s alpha of the MSPSS for the original study was 0.91 for the significant others subscale, 0.87 for the family subscale, 0.85 for the friends subscale, and 0.88 for the total scale (14). Cronbach’s alpha of the MSPSS of the present study sample was 0.96 for the significant others subscale, 0.89 for the family subscale, 0.96 for the friends subscale, and 0.82 for the total scale. The item-total score correlations ranged from 0.36 to 0.67, with an average of 0.54. Cronbach’s alpha of the MSPSS for the present study sample was 0.82. The item-total score correlations were found as 0.48 for MSPSS family, 0.89 for MSPSS friends, and 0.06 for MSPSS significant others with an average of 0.48.

### 2.2. Statistical analysis

All statistical analyses were performed by using SPSS (Armonk, NY, United States: IBM Corp.) version 26.0. The analysis and presentation of categorical variables in the form of frequencies and percentages were done. Mean and standard deviation were used to display the continuous variables. Since the data were non-normally distributed, Spearman’s rank order test was used for correlation analyses. Hierarchical regression analyses were performed to examine the predictive relationship between social support and sleep quality parameters. A value of p of less than 0.05 and 0.01 was accepted as statistically significant.

## 3. Results

The average age of 200 participants in the study was 52.3 with a standard deviation of 14.13, and it ranged from 18 to 68. The sample consisted of women (41.5%) and 117 men (58.5%) undergoing hemodialysis. The majority of the participants in the study were married (*n* = 139, 69.5%) and 6% were single (*n* = 12), and the remaining participants (*n* = 49, 24.5%) were either divorced or widowed. The majority of the participants in the study were illiterate (*n* = 137, 68.5%), 84% of the participants had a duration of CKD of 1–5 years, and 88.5% had a duration of HD of 1–5 years. In 58.5% of participants, hypertension was reported as the cause of ESRD, and in 20% of the participants, diabetes mellitus was reported as the cause of ESRD (see Table 1).

**Table 1:** Sociodemographic characteristics of the study participants (*n* = 200).

Variable	Category	n	%
Age (years)	18–24	9	4.5
	25–34	22	11
	35–44	28	14
	45–54	45	22.5
	> 55	96	48
Gender	Female	83	41.5
	Male	117	58.5
Marital status	Single	12	6
	Married	139	69.5
	Divorced	24	12
	Widowed/Widower	25	12.5
Education status	Illiterate	137	68.5
	Intermediate	20	10
	Secondary	30	15
	University	13	6.5
Occupational status	Employed	188	94
	Unemployed	11	5.5
	Retired	1	0.5
Family income	Unknown	88	44
	1,000–1,500 dollars	22	11
	1,500–2000 dollars	45	22.5
	> 2000 dollars	45	22.5
Duration of CKD	< 1 year	40	20
	1–3 years	69	34.5
	3–5 years	59	29.5
	> 5 years	32	16
Cause of ESRD	Hypertension	117	58.5
	Diabetes mellitus	40	20
	Glomerulonephritis	5	2.5
	Others	38	19
Duration on hemodialysis	3 months	25	12.5
	1 year	46	23
	1–3 years	51	25.5
	3–5 years	55	27.1
	> 5 years	23	11.5
Number of dialysis sessions per week	Once a week	21	10.5
	Twice a week	140	70
	Thrice a week	34	17
	Four times a week	5	2.5

CKD: chronic kidney disease. ESRD: end-stage renal disease.

Sixty-three subjects (31.5%) reported poor sleep quality defined as a total PSQI score > 5. Forty-one subjects (20.5%) reported clinically significant (moderate-to-severe) insomnia defined as with a total ISI score > 14. The mean PSQI total score was 4.39 (SD 4.73), the mean ISI total score was 5.81 (SD 7.65), the mean MSPSS family score was 6.55 (SD 0.81), the mean MSPSS friends’ score was 2.72 (SD 1.87), the mean MSPSS significant others score was 1.10 (SD 0.55), and the mean MSPSS total score was 3.45 (SD 0.76; see Table 2).

**Table 2:** Descriptive characteristics for the PSQI, ISI, and MSPSS (*n* = 200).

Variable	Mean	Std. Deviation	Minimum	Maximum
PSQI	4.39	4.73	0	16
ISI	5.81	7.65	0	25
MSPSS				
MSPSS family	6.55	0.81	2.75	7
MSPSS friends	2.72	1.87	1	6.75
MSPSS significant others	1.1	0.55	0.75	6.25
MSPSS total	3.45	0.76	1.75	6.33

PSQI: Pittsburgh Sleep Quality Index. ISI: Insomnia Severity Index. MSPSS: Multidimensional Scale of Perceived Social Support.

The Insomnia Severity Index total was significantly correlated with MSPSS friends (*r_s_* = −0.195, *p* < 0.01), MSPSS total (*r_s_* = −0.159, *p* < 0.05), and PSQI total (*r_s_* = 0.083, p < 0.05). PSQI total was negatively correlated with MSPSS friends (*r_s_* = −0.294, *p* < 0.01), and MSPSS total (*r_s_* = −0.222, *p* < 0.01). MSPSS total was significantly correlated with MSPSS family (*r_s_* = 0.481, *p* < 0.01), MSPSS friends (*r_s_* = 0.892, *p* < 0.01), and family income (*r_s_* = 0.212, *p* < 0.01). MSPSS family was significantly correlated with MSPSS friends (*r_s_* = 0.145, *p* < 0.05), family income (*r_s_* = 0.231, *p* < 0.01), and the duration of CKD (*r_s_* = −0.140, *p* < 0.01). MSPSS friends was significantly correlated with age (*r_s_* = 0.177, *p* < 0.05), and family income (*r_s_* = 0.177, *p* < 0.05). MSPSS significant others significantly correlated with MSPSS family (*r_s_* = −0.132, *p* < 0.63; see Table 3).

**Table 3:** Correlation between perceived social support measures and demographics and scales.

	1	2	3	4	5	6
ISI total	1					
PSQI total	−0.803^**^	1				
MSPSS total	−0.159^*^	−0.222^**^	1			
MSPSS family	0.091	0.088	0.481^**^	1		
MSPSS friends	−0.195^**^	−0.294^**^	0.892^**^	0.145^*^	1	
MSPSS significant others	−0.035	−0.007	0.060	−0.132	0.044	1
Age	0.088	0.127	−0.126	0.057	−0.167^*^	−0.124
Duration of CKD	0.115	0.136	−0.126	−0.140^*^	−0.102	0.028
Family income	0.094	0.011	0.212^**^	0.231^**^	0.177^*^	−0.086

^*^p < 0.05; ^**^p < 0.01. ISI: Insomnia Severity Index. PSQI: Pittsburgh Sleep Quality Index. MSPSS: Multidimensional Scale of Perceived Social Support. CKD: Chronic kidney disease.

In hierarchical regression analyses, MSPSS friends was a significant predictor of ISI total (*β* = −0.261, *t* = −3.545, *p* = 0.000). MSPSS total score was a significant predictor of ISI total (*β* = −0.167, *t* = −2.253, *p* = 0.025). Approximately, 11.5% of variability in PSQI total scores and 8.5% increase in predictive capacity were accounted for the inclusion of MSPSS significant others, MSPSS family, and MSPSS friends subscores [*F*(3, 191) = 6.110, *p* = 0.001]. MSPSS family score was a significant predictor of PSQI total (*β* = 0.142, *t* = 2.007, *p* = 0.046). MSPSS friends score was a significant predictor of PSQI total (*β* = −0.286, *t* = −3.926, *p* = 0.000; see Table 4A). MSPSS total score was a significant predictor of PSQI total (*β* = −0.183, *t* = −2.487, *p* = 0.014). MSPSS family score was a significant predictor of subjective sleep quality (*β* = 0.142, *t* = 1.988, *p* = 0.048). MSPSS friends score was a significant predictor of subjective sleep quality (*β* = −0.294, *t* = −3.999, *p* = 0.000). MSPSS total score was a significant predictor of subjective sleep quality (*β* = −0.174, *t* = −2.335, *p* = 0.021). MSPSS friends score was a significant predictor of sleep latency (*β* = −0.227, *t* = −3.082, *p* = 0.002). Family income was a significant predictor of sleep duration (*β* = 0.149, *t* = 2.107, *p* = 0.036). MSPSS family was a significant predictor of sleep duration (*β* = 0.156, *t* = 2.248, *p* = 0.026). MSPSS friends’ score was a significant predictor of sleep duration (*β* = −0.250, *t* = −3.492, *p* = 0.001). MSPSS total score was a significant predictor of sleep duration (*β* = −0.171, *t* = −2.364, *p* = 0.019). Age was a significant predictor of sleep efficiency (*β* = 0.144, *t* = 2.000, *p* = 0.047). Gender was a significant predictor of sleep efficiency (*β* = −0.173, *t* = −2.453, *p* = 0.015). The duration of CKD was a significant predictor of sleep disturbance (*β* = 0.396, *t* = 3.162, *p* = 0.002). The duration of HD was a significant predictor of sleep disturbance (*β* = −0.306, *t* = −2.441, *p* = 0.016). MSPSS friends score was a significant predictor of sleep disturbance (*β* = −0.167, *t* = −2.256, *p* = 0.025). MSPSS friends score was a significant predictor of daytime dysfunction (*β* = −0.277, *t* = −3.748, *p* = 0.000). MSPSS total score was a significant predictor of daytime dysfunction (*β* = −0.195, *t* = −2.626, *p* = 0.009; see Table 4B).

**Table 4A:** A Hierarchical regression analyses of MSPSS dimensions.

Model	Independent variables	*B*	*t*	*p*	*F*	*df*	*R* ^2^	Model *p*
1	MSPSS Friends	−0.261	−3.545	0.000	5.018	3, 191	0.097	0.002
	MSPSS Total score	−0.167	−2.253	0.025				
2	MSPSS Family	0.142	2.007	0.046	6.110	3, 191	0.115	0.001
	MSPSS Friends	−0.286	−3.926	0.000				
	MSPSS Total score	−0.183	−2.487	0.014				

Model 1: Dependent variable ISI total score. Model 2: Dependent variable PSQI total score. MSPSS: Multidimensional Scale of Perceived Social Support.

**Table 4B:** Hierarchical regression analyses of PSQI dimensions.

Model	Independent variables	*B*	*t*	*p*	*F*	*df*	*R* ^2^	Model *p*
1	MSPSS Family	0.142	1.988	0.048	6.302	3, 191	0.097	0.000
	MSPSS Friends	−0.294	−3.999	0.000				
	MSPSS Total score	−0.174	−2.335	0.021				
2	MSPSS Friends	−0.227	−3.082	0.002	3.640	3, 191	0.094	0.014
3	Family Income	0.149	2.107	0.036	5.755	3, 191	0.142	0.001
	MSPSS Family	0.156	2.248	0.026				
	MSPSS Friends	−0.250	−3.492	0.001				
	MSPSS Total score	−0.171	−2.364	0.019				
4	Age	0.144	2.000	0.047	1.780	3, 191	0.093	0.152
	Gender	−0.173	−2.453	0.015				
5	Duration of CKD	0.396	3.162	0.002	1.769	3, 191	0.086	0.154
	Duration of HD	−0.306	−2.441	0.016				
	MSPSS Friends	−0.167	−2.256	0.025				
6	MSPSS Friends	−0.277	−3.748	0.000	5.108	3, 191	0.087	0.002
	MSPSS Total score	−0.195	−2.626	0.009				

Model 1: Dependent variable subjective sleep quality. Model 2: Dependent variable sleep latency. Model 3: Dependent variable sleep duration. Model 4: Dependent variable sleep efficiency. Model 5: Dependent variable sleep disturbance. Model 6: Dependent variable daytime dysfunction. PSQI: Pittsburgh Sleep Quality Index. MSPSS: Multidimensional Scale of Perceived Social Support. CKD: Chronic kidney disease. HD: Hemodialysis.

## 4. Discussion

This study examined the relationship between social support and the quality of sleep in a sample of Somalian patients undergoing HD. A total of 32% of patients undergoing HD were poor sleepers with a total PSQI score > 5. Approximately, 30% of patients reported insomnia defined as having a total ISI score of > 7, and 21% of patients had clinically significant insomnia. While mean perceived friends’ support, significant others support, and total social support scores were lower than the original scale American sample, perceived family support scores were higher than the original scale sample (13). The majority of our patients undergoing HD reported remarkably higher family support but low friends and significant others support. Poor sleep quality (measured by the PSQI Total) significantly correlated with perceived friends’ support and perceived total social support. Insomnia severity (measured by the ISI Total) was significantly correlated with perceived friends’ support and perceived total social support. While perceived family support significantly correlated with both family income and duration of CKD, perceived friends’ support significantly correlated with age and family income. Both perceived family support and friends’ support were significant predictors of poor sleep quality. Perceived friends’ support was a significant predictor of insomnia severity. Perceived family support was a significant predictor of subjective sleep quality and sleep duration. Perceived friends’ support was a significant predictor of subjective sleep quality, sleep duration, sleep latency, sleep disturbance, and daytime dysfunction. Family income was a significant predictor of sleep duration. Both age and gender were significant predictors of sleep efficiency. Both the duration of CKD and the duration of HD were significant predictors of sleep disturbance.

The results of the present study showed that the prevalence of poor sleepers in patients undergoing HD was remarkably lower than in previous reports, ranging from 71 to 83.8% in other studies (15–18). Approximately 30% of patients reported insomnia, from which 21% had clinically significant insomnia. This was lower than the 55% that (19) reported earlier. The results of the present study also showed that Somalian patients undergoing hemodialysis had high perceived social support from their family and poor perceived social support from their friends. There are no established population norms on perceived social support. Norms would likely vary on the basis of culture and nationality, as well as age and gender. Overall, our findings were consistent with the traditional collective culture of the Somalian nation and validate the value of family as the principal support system in Somalian culture.

Our results showed that poor sleep quality and insomnia severity were negatively correlated with perceived friends and total social support. While perceived family support positively correlated with family income, it was negatively correlated with the duration of CKD. As the duration of CKD increased, family support levels decreased. Similarly, perceived friends’ support positively correlated with family income and negatively correlated with age. As the family income increased, friends’ support levels increased and the older patients had significantly worse friends’ support than younger patients.

While perceived family support was a significant predictor of subjective sleep quality and sleep duration, perceived friends’ support was a significant predictor of subjective sleep quality, sleep duration, sleep latency, sleep disturbance, and daytime dysfunction. Family income was a significant predictor of sleep duration. This finding was consistent with the notion that a sufficient amount of family income to afford living and treatment expenses played a crucial role equivalent to physical factors to contribute to the overall quality of life (19, 20). Both age and gender were significant predictors of sleep efficiency. Both the duration of CKD and the duration of HD were significant predictors of sleep disturbance. Our findings revealed that perceived family and friends’ support were significant predictors of overall sleep quality. These results were intuitive since the majority of our patients undergoing HD had no job or fund, and they could only afford their required hemodialysis sessions by relying on the family as their primary social and financial resource, and their access to quality care was extremely limited. Our results were consistent with previous reports and supported the notion that the higher level of perceived family and friends’ support helped the patients to adapt to their chronic diseases physically and mentally and improved their coping with the chronic disease and treatments (10, 21–23). Our findings also documented further evidence that perceived social support could be an essential component of coping mechanism in a better adaptation to the burden of having end-stage renal disease and could play a fundamental role in enhancing the physical and mental health of the patients undergoing hemodialysis. A fundamental principle of social relations in Somalian society is the principle of collective responsibility. The individual is surrounded by his/her family, in the second circle matrilateral and patrilateral relatives, half-siblings, close friends, etc. Although close kin relations do not necessarily entail closer social relations, the constitution of Somalian society lies first in kinship and family, and this has been integrated into the DNA of Somalian social relations. Family provides stability and longevity to Somalian society as an agreed set of social practices and contributes to stable social relations. In contrast, friendships in the psychosocial reality of Somalian society do not follow the structure in Western societies and are entirely formal and hierarchical at the same time. Therefore, the driving force of social relations which forms Somalian communities is predominantly family-based which is so well attuned to traditional Somalian life.

This study has certain limitations. The assessment of the potential effects of biochemical laboratory parameters could have been more informative. A control group of patients including pre-dialysis stage patients with ESRD could have provided comparative data, and this can be accomplished in a follow-up study. Despite these, the findings of the present study for the first time reported the status of Somalian patients undergoing hemodialysis and might enhance our team’s efforts to convince Somalian government agencies to plan better hemodialysis services in the near future.

In conclusion, the present study has highlighted for the first time the associated factors causing poor sleep and the impact of perceived social support on the quality of sleep in a sample of patients undergoing HD in Somalia. The present study contributed to the limited research knowledge that examined the relationship between social support and the quality of sleep of patients undergoing hemodialysis in Africa. Patients undergoing hemodialysis might benefit from receiving formal and informal social support such as support from family and friends. Healthcare professionals working in hemodialysis units should continuously assess patients’ quality of sleep and monitor the level of social support to improve their treatment adherence. Healthcare policymakers should consider social support as a high-priority area of work and research to enhance the management of patients undergoing hemodialysis.

## Data availability statement

The raw data supporting the conclusions of this article will be made available by the authors, without undue reservation.

## Ethics statement

The studies involving human participants were reviewed and approved by Mogadishu Somalia Turkey Recep Tayyip Erdogan Research and Training Hospital Ethics Review Board (MSTH/10515, Date: 05/30/2022). The patients/participants provided their written informed consent to participate in this study.

## Author contributions

SK: guarantor of integrity of the entire study. SK and NM: study concepts and designs, statistical analysis. SK, NM, YM, and AE: literature research and manuscript preparation. All authors contributed to the article and approved the submitted version.

## Conflict of interest

The authors declare that the research was conducted in the absence of any commercial or financial relationships that could be construed as a potential conflict of interest.

## Publisher’s note

All claims expressed in this article are solely those of the authors and do not necessarily represent those of their affiliated organizations, or those of the publisher, the editors and the reviewers. Any product that may be evaluated in this article, or claim that may be made by its manufacturer, is not guaranteed or endorsed by the publisher.
